# Early urinary biomarkers of diabetic nephropathy in type 1 diabetes mellitus show involvement of kallikrein-kinin system

**DOI:** 10.1186/s12882-017-0519-4

**Published:** 2017-03-30

**Authors:** Lenka Vitova, Zdenek Tuma, Jiri Moravec, Milan Kvapil, Martin Matejovic, Jan Mares

**Affiliations:** 1 Department of Internal Medicine, Teaching Hospital Motol, V Uvalu 84, Prague, 5, 150 06 Czech Republic; 2 Proteomic Laboratory, Charles University School of Medicine in Pilsen, alej Svobody 1655/76, Pilsen, 323 00 Czech Republic; 3 Department of Internal Medicine I, Charles University School of Medicine in Pilsen, alej Svobody 80, Pilsen, 304 60 Czech Republic

**Keywords:** Diabetes, Nephropathy, Proteomics, Kallikrein-kinin system

## Abstract

**Background:**

Additional urinary biomarkers for diabetic nephropathy (DN) are needed, providing early and reliable diagnosis and new insights into its mechanisms. Rigorous selection criteria and homogeneous study population may improve reproducibility of the proteomic approach.

**Methods:**

Long-term type 1 diabetes patients without metabolic comorbidities were included, 11 with sustained microalbuminuria (MA) and 14 without MA (nMA). Morning urine proteins were precipitated and resolved by 2D electrophoresis. Principal component analysis (PCA) and Projection to latent structures discriminatory analysis (PLS-DA) were adopted to assess general data validity, to pick protein fractions for identification with mass spectrometry (MS), and to test predictive value of the resulting model.

**Results:**

Proteins (*n =* 113) detected in more than 90% patients were considered representative. Unsupervised PCA showed excellent natural data clustering without outliers. Protein spots reaching Variable Importance in Projection score above 1 in PLS (*n =* 42) were subjected to MS, yielding 33 positive identifications. The PLS model rebuilt with these proteins achieved accurate classification of all patients (R2X = 0.553, R2Y = 0.953, Q2 = 0.947). Thus, multiple earlier recognized biomarkers of DN were confirmed and several putative new biomarkers suggested. Among them, the highest significance was met in kininogen-1. Its activation products detected in nMA patients exceeded by an order of magnitude the amount found in MA patients.

**Conclusions:**

Reducing metabolic complexity of the diseased and control groups by meticulous patients’ selection allows to focus the biomarker search in DN. Suggested new biomarkers, particularly kininogen fragments, exhibit the highest degree of correlation with MA and substantiate validation in larger and more varied cohorts.

**Electronic supplementary material:**

The online version of this article (doi:10.1186/s12882-017-0519-4) contains supplementary material, which is available to authorized users.

## Background

Diabetic nephropathy (DN) is a major complication of diabetes mellitus (DM), largely responsible for the outbreak of dialysis-dependent end stage renal disease we have witnessed during last few decades. It substantially impairs quality of life in patients with diabetes and imposes a considerable burden of health-care costs [1].

Once developed, DN cannot be efficiently reversed; hence successful management must be based on timely selection of patients at risk and therefore indicated to more stringent glycaemic control. A prerequisite of such approach is availability of a non-invasive, highly sensitive and specific screening tool [2]. Currently, this task is usually achieved by regular measurement of urinary albumin excretion. Nevertheless, microalbuminuria (MA) is neither a specific nor an early marker and misdiagnosis or underestimation of evolving DN often undermines treatment outcomes [3]. Therefore, some authors suggest leaving this concept completely [4]. So obviously we need more precise criteria to define target populations which would benefit from the emerging strategies for diabetes therapy.

Unlike MA, which roughly estimates glomerular permeability, new biomarkers promise to reflect more aspects of DN and hence to detect earlier stages with enhanced specificity [5]. Mass spectrometry-based (MS) proteomics provides an unbiased and complex approach to analysis of protein mixtures which seems optimal for this task [6] and several attempts have been made to define a specific urinary proteome in patients with DN or even to discover more reliable biomarkers. However, most of these studies have serious limitations and so far no markers were introduced into clinical practice [7]. Apparently, such a goal cannot be reached by a single study – first, potential biomarkers must be searched for in limited, well defined groups of patients and consequently validated on larger populations [6].

As validation trials are cost and time consuming, studies searching for new markers of DN must be carefully designed and address several challenges: a study group with maximum homogeneity and appropriate controls, a preparation method covering wide range of molecular weights and yielding enough material for identification, and MS technology capable to describe both intact proteins and fragments. Here we present a discovery-phase trial based on these requirements.

## Methods

### Study design and patient population

This was a prospective, observational, controlled, single-centre study. The study protocol was approved by Ethics Committee for Multi-Centric Clinical Trials of the University Hospital Motol (ref. No EK-1407/08); all subjects signed an informed consent before enrolment in the study. Patients were recruited at Motol University Hospital (Prague, Czech Republic) outpatient clinic, primarily through database search; the selection criteria were subsequently validated on the screening visit.

The inclusion criteria were as follows: age >18 years, ability to sign informed consent, type 1 DM confirmed by C peptide with minimum duration of 15 years, BMI <30 kg/m^2^, and at least 3 follow-ups including albumin excretion test within last year. Exclusion criteria were as follows: history of kidney disease (other than DN), estimated glomerular filtration rate (eGFR) <60 ml/min/m^2^, active infection, malignancy, liver disease, or cardiopulmonary insufficiency.

Patients showing persistent microalbuminuria (defined as 2.5-35 mg/mmol albumin/creatinine ratio or albuminuria 30–300 mg/24 h on at least 2 of 3 follow-ups) and diabetic retinopathy were assigned to the study group (MA). The control group (nMA) was compiled of patients suffering from type 1 DM for over 20 years and showing no microalbuminuria (or retinopathy) on the last three follow-ups (or previously) even without any type of renin-angiotensin system blockade.

Totally, 32 patients were enrolled yet only 25 of them were analysed – in 5 patients criteria of MA were not met at the study visit and in two control subjects urine samples did not yield enough protein for two-dimensional electrophoresis (2-DE). Renin-angiotensin-aldosterone system (RAAS) antagonists were discontinued per protocol one day before sample collection. The period of 24 h has been chosen to ensure maximum reduction in the direct RAAS inhibition without wasting its long-term benefits and inflicting structural damage.

### Clinical and laboratory evaluation

Patients’ demographic and clinical characteristics including medical history, current and past medication, height, weight, and blood pressure were obtained by interview, examination, and medical record review on the day of the screening visit. The first morning urine was collected for protein analysis. Blood and urine samples were drawn and sent for routine laboratory analysis including serum creatinine, glycated haemoglobin, and lipid levels, urinary creatinine and albumin concentrations. eGFR was calculated using CKD-EPI formula.

### Urinary protein preparation

All chemicals used during preparation and analyses were purchased from Sigma (Sigma-Aldrich, Steinheim, Germany).

All urine samples were processed within 4 h after collection. The mean processed sample volume was 156 ml in MA patients and 246 ml in nMA patients. Mean protein concentration (determined with Bradford assay) was 320 mg/l (±103.2 mg/l) in MA group and 50.1 mg/l (±6.7 mg/l) in nMA group. Each sample was first centrifuged for 15 min at 3780 g at 4 ° C to remove cells and insoluble debris. Then, the supernatant was mixed with 60% trichloroacetic acid (ratio 5:1) and incubated overnight in the ice cold water bath. On the next day, samples were centrifuged for 1 h at 3780 g at 4 °C. The pellet was washed with 5–10 ml ice-cold acetone, incubated 10 min in the ice-cold water bath, and then centrifuged for another hour. The final pellet was resuspended in the lysis buffer (7 M urea, 2 M thiourea, 4% CHAPS, 2% IPG buffer pH 3–10, 120 mM DTT, 40 mM Tris base) and stored frozen at −80 °C.

### Two-dimensional electrophoresis

The method was described in detail previously [8]. Briefly, 200 μg proteins were applied onto 11 cm IPG strips, rehydrated, and focused. The second-dimension separation was performed on precast polyacrylamide gels. Next, the gels were stained with Simply Blue and the spots of interest excised using a spot-cutter.

### MALDI (matrix-assisted laser desorption/ionization) TOF (time-of-flight) tandem MS and protein identification

The method was described in detail previously [8]. Briefly, proteolytic peptides obtained by tryptic digestion of the excised proteins were analyzed using a 4800 MALDI TOF/TOF Analyzer (Applied Biosystems, Framingham, MA, USA). Protein identification was achieved by searching against the human subset of the SwissProt protein database (Swiss Institute of Bioinformatics, Basel, Switzerland) (release 2010_09; 10 Aug 2010) using the MASCOT 2.1.0 search algorithm (Matrix Science, London, UK).

### 2-DE pattern analysis, statistics, and data presentation

Computer aided analysis of 2-DE gel images was carried out using PDQuest 2D software version 8.1 (Bio-Rad, Hercules, CA, USA). A synthetic image was constructed out of the triplicated or duplicated gels (as applicable with respect to the amount of protein available) processed from each sample, using only spots constantly present in at least two gels. The protein quantity was determined relative to integrated spot density excluding saturated spots.

The statistical analysis was carried out by means of Statistica data analysis software (Version 8.0, Statsoft Inc., Tulsa, OK, USA). Proteomic data are characterized by high degree of collinearity, multiple variable interactions, and variable numbers exceeding that of cases. Under such conditions, the traditional univariate approach fails and multivariate techniques should be considered, which allow for inter-variable correlations and reduce data dimensionality by extracting principal components (latent variables). Among them, Principal Component Analysis (PCA) is a method appropriate for unsupervised data exploration while Projection to Latent Structures – Discriminant Analysis (PLS-DA) can be used to evaluate classification potential of the data set to discriminate between predefined classes [9]. Variable Importance in the Projection (VIP) score serves to rank variables according to their significance in the PLS-DA model.

In the current study, PCA based on covariance matrix was applied to examine natural data clustering and to screen for potential outliers. Only spots detectable in > 90% samples in any of the two groups were considered eligible for PCA. PLS-DA models were built with a binary coded dummy response matrix; using 7-fold cross-validation strategy to avoid over fitting. Protein spots with regression coefficients for which the jack-knifed 95% confidence interval did not include 0 and VIP scores exceeded 1 were selected for identification and the model was re-calculated thereafter using these variables. Mann–Whitney rank sum test was employed to compare group characteristics and as a confirmatory tool for proteomic data. Differences were considered statistically significant if the p-values were < 0.01 for proteomic data and < 0.05 for other data types.

## Results

Patients’ demographic and relevant clinical characteristics are shown in Tab. 1. The two groups were homogenous with respect to gender, age, DM duration, BMI, blood pressure, and lipid profile. Not surprisingly, patients with MA had higher glycated haemoglobin level and slightly worse kidney function as indicated by serum creatinine and eGFR. Clearly, urinary albumin/creatinine ratio was markedly higher in the MA group.

**Table 1:** Study groups – demographic and clinical characteristics

	microalbuminuric	non-microalbuminuric	p
Cases (male/female)	11 (6/5)	14 (6/8)	0.57
DM duration (years)	26 (20–30)	22.5 (21–25)	0.78
Age (years)	35 (33–44)	35 (32–40)	0.76
BMI (kg/m^2^)	25.35 (21.97–28.6)	24.15 (23.51–24.96)	0.41
Systolic BP (mmHg)	138 (118–154)	138 (124–143)	1
Diastolic BP (mmHg)	84 (77–88)	79.5 (74–90)	0.62
Serum creatinine (μmol/l)	87 (81–116)	71.5 (61–76)	<0.01
eGFR (ml/min)^a^	77.8 (63.6–100.4)	108.8 (101.5–113.1)	<0.01
Albumin/creatinine ratio (mg/mmol)^b^	12.17 (5,56–15.15)	0.505 (0.43–0.7)	<0.0001
HbA1c	7.9% (6.9–8.5) 63 mmol/mol (52–69)	6% (5.3–7.1) 42 mmol/mol (34–54)	<0.01
Serum cholesterol (mmol/l)	4.6 (4.1–5.4)	5.4 (4.5–5.7)	0.13
Triglycerides (mmol/l)	1.21 (1.09–1.34)	0.88 (0.72–1.97)	0.38

Data are given as medians (IQR), statistical significance was confirmed with Mann–Whitney rank sum test

^a^eGFR was calculated by CKD-EPI formula

^b^In 9 non-microalbuminuric patients, urinary albumin was under the detection limit (<3 mg/l), here the albumin concentration was set arbitrarily to 3 mg/l

**Fig. 1:**
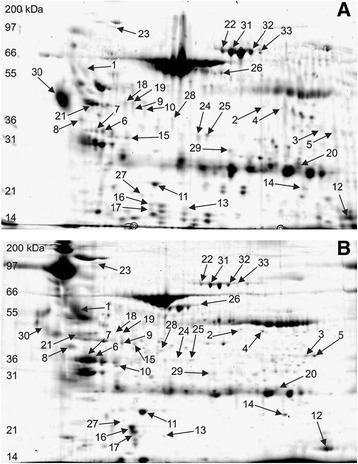
Representative gel images from individual patients with (**a**) and without (**b**) microalbuminuria. Spot numbers correspond with the list in Table 2

**Fig. 2:**
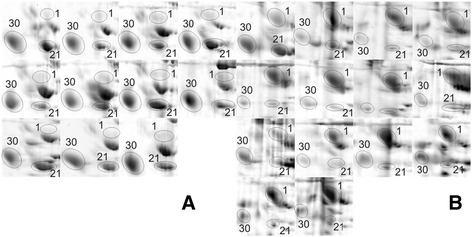
Kininogen-1 in patients with (**a**) and without (**b**) microalbuminuria. Magnified areas of 2D gels from all patients show Kininogen-1 activation product (heavy chain, spot #1), Alpha-1-acid glycoprotein 1 (spot #30), and Zinc-alpha-2-glycoprotein (spot #21)

Two-dimensional electrophoresis (2DE) of purified urinary protein yielded a total of 755 protein fractions (spots). Of this number, 100 spots were detected exclusively in the MA group, 88 solely in the nMA group, and 567 were documented in both. Individual gels (one from each group) with most typical spot distribution, i.e. containing most of the detected spots, are presented in Fig. 1 and a magnified area in Fig. 2. Spots detected in more than 90% gels in any group (*n =* 113) were arbitrarily considered representative (of either group) and as such included into the PCA. The projection of individual cases on the factor plane is shown in Additional file 1. The first principal component separated all patients into two compact clusters leaving no obvious outliers. Moreover, these two naturally occurring clusters precisely agreed with the study groups divided according to microalbuminuria.

**Fig. 3:**
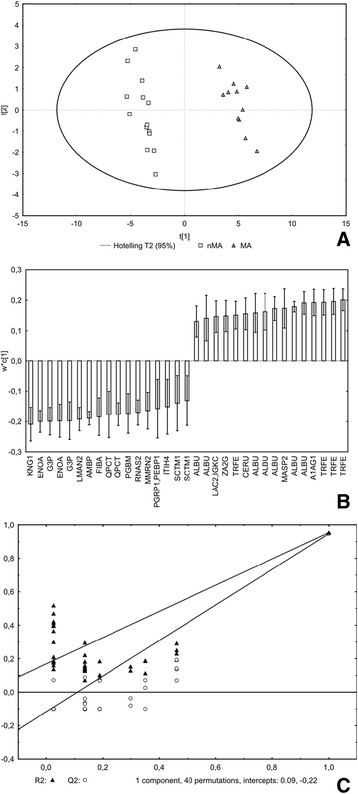
Partial least squares – discriminant analysis: final model representing 33 protein fractions. panel **a**: scores plot (shows supervised projection of all cases). panel **b**: loadings plot (shows contribution of variables to classification, positive are surplus in MA group and vice versa). panel **c**: validation plot (shows superiority of suggested model over random models). R2X = 0.553, R2Y = 0.953, Q2 = 0.947. VIP = Variable Importance in Projection, MS = mass spectrometry

To directly test the performance of specific proteins in assigning patients to the MA and nMA groups, PLS-DA model was built on the 113 spot intensities. Variables (*n =* 49) reaching VIP score > 1.0 were regarded to have the highest discriminatory power and therefore apt for further analysis (Additional file 2). During manual review, 7 spots were sorted out for being either inadequately resolved (e.g. overlapping with adjacent spots) or too faint to guarantee successful MS identification. Remaining 42 spots were subjected to MS/MS which was accomplished in 33 spots. Overall, 23 individual proteins were identified – their description as well as differences in abundance between both groups are given in Tab. 2. Both PLS-DA models (i.e. based on 113 and 33 variables) were capable to correctly classify all patients (positive as well as negative predictive value = 100%); detailed characteristics of the latter model are shown in Fig. 3 (R2X = 0.553, R2Y = 0.953, Q2 = 0.947).

**Table 2:** Proteins differentiating between microalbuminuric and non-microalbuminuric groups

				Theoretical^a^		MS		MS/MS			
Spot No	Protein Name	SwissProt entry	Biological function^b^	MW (kDa)	pI	Peptide count	Protein score	Peptide count	Total ion score	Spot intensity ratio^c^	p^d^
1	Kininogen-1	KNG1	blood coagulation, source of bradykinin	71912	6,34			5	184	0.110	<0,0001
2	Alpha-enolase	ENOA	glycolysis (step 9/10), cell growth, fibrinolysis	47139	7,01			4	106	0.076	<0,0001
3	Glyceraldehyde-3-phosphate dehydrogenase	G3P	glycolysis (step 6/10), nuclear regulatory functions, apoptosis	36030	8,57			2	57	0.018	<0,0001
4	Alpha-enolase	ENOA		47139	7,01	6	57	5	257	0.140	<0,0001
5	Glyceraldehyde-3-phosphate dehydrogenase	G3P		36030	8,57			1	70	0.091	<0,0001
6	Vesicular integral-membrane protein VIP36	LMAN2	intracellular protein trafficking (Golgi to ER)	40203	6,46			2	81	0.335	<0,001
7	Protein AMBP	AMBP	trypsin inhibitor	38974	5,95			1	35	0.331	<0,01
8	Fibrinogen alpha chain	FIBA	blood coagulation	94914	5,7			2	40	0.238	<0,001
9	Glutaminyl-peptide cyclotransferase	QPCT	N-terminal pyroglutamate formation	40851	6,12	8	67	4	161	0.165	<0,001
10	Glutaminyl-peptide cyclotransferase	QPCT		40851	6,12			3	99	0.190	<0,0001
11	Basement membrane-specific heparan sulphate proteoglycan core protein	PGBM	component of basement membrane, anti-angiogenesis	468501	6,06			5	347	0. 364	<0,001
12	Non-secretory ribonuclease	RNAS2	non-secretory ribonuclease	18342	9,1			5	391	0.162	<0,001
13	Multimerin-2	MMRN2	endothelial surface protein, anti-angiogenesis	104344	5,56			1	51	0.202	<0,01
14	Peptidoglycan recognition protein 1	PGRP1	pattern recognition, innate immunity	21717	8,92			2	122	0.366	<0,01
14	Phosphatidylethanolamine-binding protein 1	PEBP1	serine protease inhibitor, Raf kinase inhibitor	21044	7,01			1	58		<0,01
15	Inter-alpha-trypsin inhibitor heavy chain H4	ITIH4	immunomodulation (acute phase)	103293	6,51			2	46	0.406	<0,01
16	Secreted and transmembrane protein 1	SCTM1	T-cell co-stimulatory ligand	27022	7			2	93	0.219	<0,01
17	Secreted and transmembrane protein 1	SCTM1		27022	7			1	37	0.156	<0,001
18	Serum albumin	ALBU	major plasma protein	69321	5,92			2	82	1.9	<0,001
19	Serum albumin	ALBU		69321	5,92			2	63	4.4	<0,01
20	Ig lambda-2 chain C region	LAC2	immunoglobulin	11287	6.92			2	188	3.1	<0,0001
20	Ig kappa chain C region	IGKC	immunoglobulin	11602	5.58			1	44		<0,05
21	Zinc-alpha-2-glycoprotein	ZA2G	fatty acid binding	34237	5,71	10	54	2	196	3.8	<0,0001
22	Serotransferrin	TRFE	iron binding, cell proliferation	77000	6,81	23	119	3	193	2.8	<0,01
23	Ceruloplasmin	CERU	copper binding	122128	5,44			7	495	1.2	<0,0001
24	Serum albumin	ALBU		69321	5,92			4	150	3.9	<0,001
25	Serum albumin	ALBU		69321	5,92			2	83	3.9	<0,01
26	Serum albumin	ALBU		69321	5,92			2	113	5.0	<0.01
27	Mannan-binding lectin serine protease 2	MASP2	complement activation (lectin pathway)	75685	5,47			7	430	4.2	<0,001
28	Serum albumin	ALBU		69321	5,92			4	95	5.6	<0,0001
29	Serum albumin	ALBU		69321	5,92			2	46	8.5	<0,0001
30	Alpha-1-acid glycoprotein 1	A1AG1	immunomodulation (acute phase)	23497	4,93			2	82	4.1	<0,001
31	Serotransferrin	TRFE		77000	6,81	22	111	4	248	11.6	<0,0001
32	Serotransferrin	TRFE		77000	6,81	23	147	8	268	3.8	<0,0001
33	Serotransferrin	TRFE		77000	6,81	26	151	5	396	5.5	NS

^a^Theoretical MW and pI were retrieved from SwissProt database and correspond to intact proteins, irrespective of post-translational modifications

^b^Descriptions of proteins’ biological functions were extracted from SwissProt database

^c^Spot intensity ratios are calculated from group averages and expressed relative to non-microalbuminuric group

^d^Median spot volumes were compared between the two groups using Mann–Whitney rank sum test

In most proteins, observed molecular weight (MW) and pI were in good agreement with the theoretical ones. However, in several cases the measured MW was markedly lower than predicted by SwissProt database. Here, the peptide size and amino acid sequence established by MS/MS experiments were investigated thoroughly to show if they corresponded with any documented functional domains or cleavage fragments. These included (spot No, measured MW, calculated MW of the fragment/intact protein): kininogen-1 heavy chain (#1, MW 57, 41/70 kDa), extracellular domain of secreted and transmembrane protein 1 (# 16, 17, MW 18, 13/24 kDa), LG3 peptide from basement membrane-specific heparan sulphate proteoglycan core protein (# 11, 23, 21/467 kDa), 35 kDa chain of inter-alpha-trypsin inhibitor heavy chain H4 (# 15, MW 35, 35/70 kDa) and C1q domain of multimerin 2 (# 13, MW 18, 14/102 kDa); complete sequences are presented in Additional file 3.

## Discussion

To discover potential early biomarkers for diabetic nephropathy, a group of type 1 diabetes patients in the initial phase of the renal disease and meeting the most stringent criteria was put together, urine samples collected, and isolated proteins resolved by 2-DE. Resulting proteome map was compared with that of a control group comprising type 1 diabetes patients without kidney involvement and protein fractions best explaining the between-group variation were identified by mass spectrometry. Both the characteristic urinary proteome (i.e. common to more than 90% patients) and its representative subset provided an accurate classification of all patients. Among the candidate biomarkers most significantly contributing to the model, kininogen fragments deserve a special attention as the kallikrein-kinin pathway is considered to play a central role in DN pathogenesis.

During the last decade, a considerable effort was spent searching early urinary markers of DN, however the outcomes lack congruity desirable to achieve before the validation phase can be undertaken [7]. A great deal of the inconsistency can be attributed to patients’ heterogeneity. From this perspective, the major strength of the current study lies in the high degree of uniformity in terms of metabolic and co-morbid conditions, a number one prerequisite for a successful biomarker discovery. Another significant advantage is the capability of MALDI approach to identify proteins from a wide range of molecular weights including fragments.

Urine has been shown to reflect many pathologic and physiologic conditions with the highest accuracy and sensitivity [5]. At the same time, diabetes is a profound metabolic disorder presenting with a variety of organ involvements and therefore maximum homogeneity in terms of renal and extrarenal disease stage, comorbidities, and treatment should be sought. Unlike type 2 DM, associated with insulin resistance and affecting metabolism in virtually all tissues, type 1 DM in non-obese adults, if adequately substituted with insulin, provides the purest available form of DN [10]. In contrast, most studies examined type 2 DM and only few were focused on type 1 (two analysed less than 10 patients, i.e. 5 and 4) [7], while disease stage and comorbidities were rarely addressed. Urine harbours a complex mixture of variably sized, partially degraded peptides and proteins in aqueous phase - a hurdle, which can be overcome by means of gel-based proteomics [11]. Unlike many previous studies done with different technologies, we were able to gather information on proteins’ identity, quantity and fragmentation.

Inevitably, the study has limitations. The sample size is small and diagnosis of DN on the basis of MA uncertain. Moreover, low patient numbers make results vulnerable to group misclassification arising from surrogate diagnosis [12]. These weaknesses are shared by most trials and probably partly responsible for ambiguous outcomes. However, molecular technologies do not permit analysing large patient groups and kidney biopsy is neither clinically relevant nor ethically justifiable “per protocol”. To minimize the chance of error, selection criteria were set to conform to the highest possible specificity. Therefore only patients with diabetic retinopathy, suffering from DM long enough to develop DN and showing sustained microalbuminuria (without erythrocyturia or overt proteinuria) were considered eligible. The major disadvantage of gel-based approach, namely the laborious procedure restricting numbers of proteins analysed, was balanced by a careful selection of spots for identification [9].

In the urine of MA patients, equally as many proteins were found down-regulated as up-regulated. This result, while in agreement with previous studies, seems counterintuitive [7]. A possible explanation would be that proteinuria reflects not only increased permeability of glomeruli but also dysfunction of various physiological systems. The proteins ranged in MW from 18 to 120 kDa and included many traditional biomarkers of DN: albumin (multiple forms), transferrin, ceruloplasmin, immunoglobulin kappa and lambda chains, α1-microglobulin, or orosomucoid [13]. Intact albumin was not included in the analysis as its quantification was hampered by spot saturation; however several albumin fragments were found up-regulated. Newly discovered proteins are both intracellular and secreted, bearing both enzymatic, signalling, and structural functions and covering all major pathways recognized in DN pathophysiology, especially metabolic derangement, inflammation, regulatory, and structural (vascular) alterations.

Although the exact function of the kallikrein-kinin system in the pathogenesis of DN has not been fully elucidated, it is considered to play a beneficial role, closely cooperating with renin-angiotensin system [14]. Presumably, it exerts its activity via nitric oxide synthesis, thus affecting kidney haemodynamics, metabolism, inflammatory processes, etc. Clinical experience has been recently supported by mechanistic experiments showing protective effects of local kininogen expression on evolution of microalbuminuria [15]. In the current study, the most significant predictor of group assignment to MA group was diminished urinary excretion of kininogen-1 heavy chain (i.e. cleavage fragment documenting prior bradykinin release).

Kidney has been established as an important site of glucose metabolism and increased oxidative stress due to impaired glycolysis is considered the major culprit responsible for development of angiopathy and organ complications [16–18]. Glycolytic flux is blocked upstream from glyceraldehyde-3-phosphate dehydrogenase (G3P) which is supposed to play a central role in the dysregulation. Subsequent diversion of glycolytic intermediates into other pathways (polyol, hexosamine) leads to excessive generation of reactive oxygen species. Decreased excretion of G3P and enolase in the MA group could reflect this condition, though both enzymes exhibit also other effects such as cell cycle regulation, apoptosis, and fibrinolysis.

DN has been further linked to low-grade inflammation and activation of the complement system. Specifically, mannan-binding lectin (MBL) levels correlate with the progression of renal disease in type 1 DM [19]. MBL together with its associated protease (mannan-binding lectin serine protease 2 - MASP2) initiates the complement cascade [20] and, indeed, the activated form of MASP2 was increased in MA group. Glutaminyl-peptide cyclotransfrase (QPCT) is a secreted protein catalyzing formation of N-terminal pyroglutamate, a post-translational modification rendering the target peptides less degradable. It has been shown an important factor affecting macrophage infiltration [21] which is in turn hold responsible for tubular and mesangial inflammation during early phase of DN [22]. Also the extracellular portion of another protein, vesicular integral-membrane protein VIP36, is involved in the regulation of macrophage phagocytosis (and shedded during the process) [23]. Finally, secreted and transmembrane protein 1 (SCTM1) is a T-cell co-stimulatory ligand produced by monocytes and neutrophils [24].

LG3 peptide derived from perlecan (basement membrane-specific heparin sulphate proteoglycan core protein), a negatively charged proteoglycan and a major constituent of glomerular basement membrane, was found depleted in urine from the MA group. Although its indispensability for the membrane’s selective properties under physiological conditions has been challenged recently [25], perlecan probably acts as a pathogenic factor in diseased states [26]. Moreover, the LG3 fragment expresses anti-angiogenic properties [27]. Inter-α-inhibitor heavy chain 4 (ITIH4) is an acute phase reactant, yet its function is largely unknown; the sequence covered by MS/MS spanned within the kallikrein-generated 35 kDa C-terminal fragment [28]. Its serum quantity has been shown to correlate with various conditions including tumours and diabetes [29]. Multimerin-2 is an endothelial cell surface protein, proposed as an important inhibitor of vasculogenesis interfering with vascular endothelial growth factor A (VEGF) [30]. At the same time, complications of diabetes including nephropathy are inherently associated with VEGF-induced vascular remodelling and dysregulated angiogenesis [31]. Phosphatidyl-ethanolamine-binding protein 1 has been firmly linked with pathological neovascularisation in cancer [32]. Its expression in mesangial cells is promoted by high glucose [33], yet its extracellular concentration was shown to decrease in proliferative diabetic retinopathy [34].

At least in two cases the results suggest an association of MA with decreased activity, local or systemic, of the kallikrein system. While the role of kininogen-bradykinin axis has been widely recognized, the significance of ITIH4 is uncertain. It may represent another pathogenic mechanism or simply reflect low activity of kallikrein proteases. The relation of both pathways (as documented by urinary levels of identical kininogen and ITIH4 fragments) to proteinuria progression under treatment with ACE inhibitors was described lately by Rocchetti et al. in patients suffering from IgA nephropathy [35]. So, rather than constituting a specific trait of DN, this finding could imply a condition, inherent or acquired, rendering the kidney more vulnerable to variable pathogenic stimuli (speculatively haemodynamic in nature) inducing proteinuria.

The initial enthusiasm associated with proteomic biomarker quest has been gradually reduced as every new trial supplied more potential molecules yet very few applicable tests resulted. Given the diversity of mechanisms involved in DN pathogenesis, it is probably unrealistic to expect one parameter (or even a set of parameters) could unequivocally confirm or rule out the disorder. Maybe we should rather aim at characterizing the unique and time-dependent interplay of metabolic, inflammatory, and repair processes operating within a specific individual. Such approach would necessitate detection of functional clusters or pathways among discovered biomarkers and their correlation with clinical and histological descriptors. On the other hand, this kind of knowledge would be superior as it could help to stratify or personalize the therapy.

## Conclusion

Reducing metabolic complexity of the diseased and control groups by meticulous patient selection together with appropriate statistical methodology allowed to focus the biomarker search in DN. In this way, we generated a compact set of potential new biomarkers exhibiting the highest degree of correlation with MA and substantiating validation in larger and more varied cohorts. These proteins could provide, upon proper validation, either separately or in concert, more reliable, timely, and detailed diagnosis of DN.

## Additional files

Additional file 1: Principal component analysis of protein spot intensities – composite projection of cases into the factor plane defined by the first two principal components (PC)”. (DOCX 23 kb)

Additional file 2: Variable importance in projection (VIP) scores for the 113 spots entering the PLS-DA model”. (DOCX 84 kb)

Additional file 3: Combined MS and MS/MS sequence coverage of detected cleavage fragments”. (DOCX 22 kb)

## Abbreviations

2-DE: Two-dimensional electrophoresis
BMI: Body mass index
CHAPS: (3-((3-cholamidopropyl) dimethylammonio) 1-propanesulfonate)
DM: Diabetes mellitus
DN: Diabetic nephropathy
DTT: Dithiothreitol
eGFR: Estimated glomerular filtration rate
IPG: Imobilized pH gradient
ITIH4: Inter-α-inhibitor heavy chain 4
MA: Microalbuminuria
MALDI: Matrix-assisted laser desorption/ionization
MASP2: Mannan-binding lectin serine protease 2
MS: Mass spectrometry
MW: Molecular weight
nMA: Without microalbuminuria
PCA: Principal component analysis
PLS-DA: Projection to latent structures discriminatory analysis
QPCT: Glutaminyl-peptide
SCTM1: Secreted and transmembrane protein 1
TOF: Time-of-flight
VEGF: Vascular endothelial growth factor A
VIP: Variable importance in the projection
VIP36: Vesicular integral-membrane protein
