# Supplementary material for: Early urinary biomarkers of diabetic nephropathy in type 1 diabetes mellitus show involvement of kallikrein-kinin system
Source: BMC Nephrol. 2017 Mar 30;18:112. doi: 10.1186/s12882-017-0519-4 (PMC5372325; doi:10.1186/s12882-017-0519-4)
Supplement: Supplementary file 1 — Principal component analysis of protein spot intensities – composite projection of cases into the factor plane defined by the first two principal components (PC)”. (DOCX 23 kb) [file 12882_2017_519_MOESM1_ESM.docx]

**
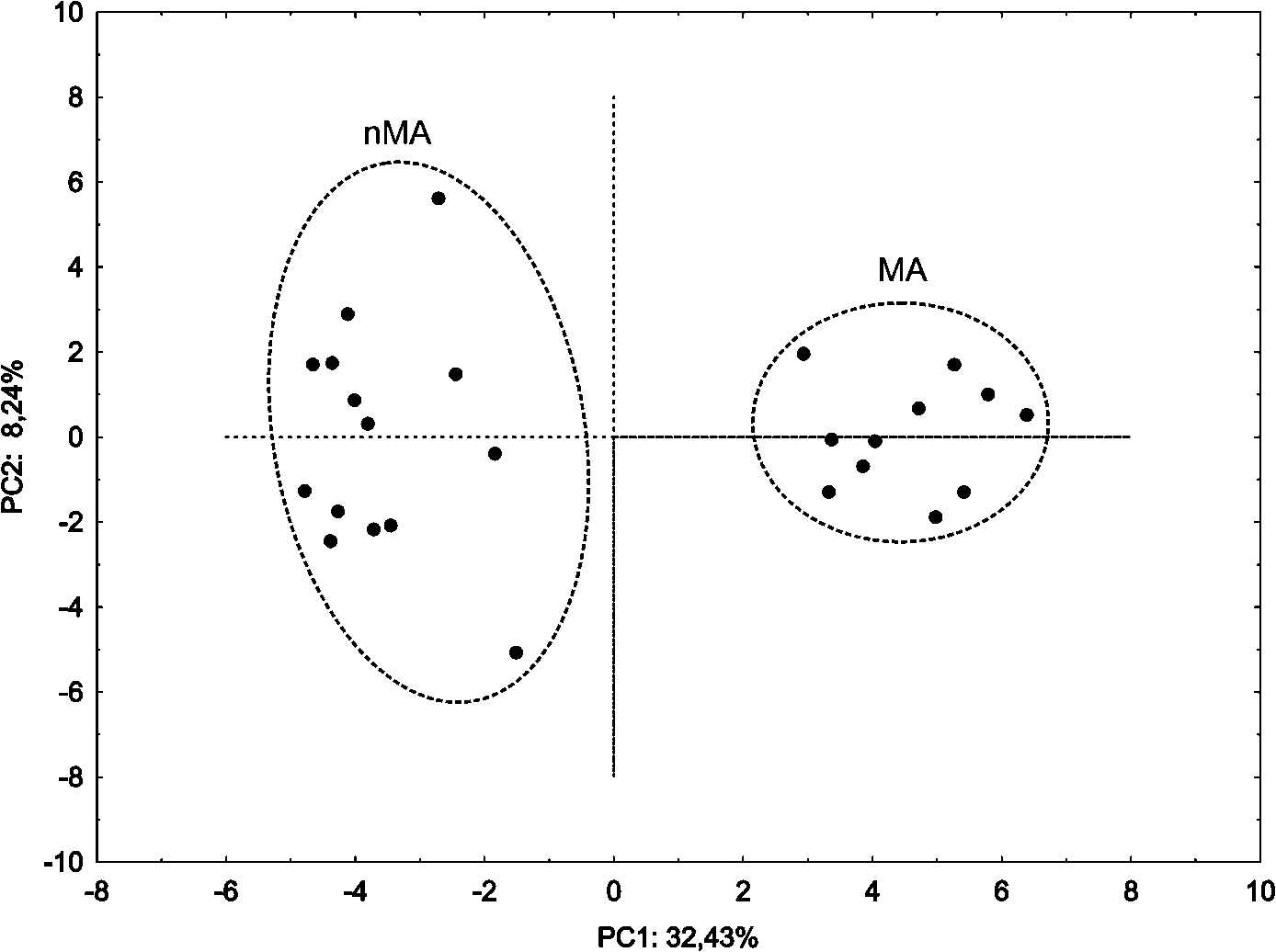
**

**Supplement 1 Principal component analysis of protein spot intensities – composite projection of cases into the factor plane defined by the first two principal components (PC)**

Patients’ assignment into the study groups is indicated by dashed ellipses.
