# Supplementary material for: Early urinary biomarkers of diabetic nephropathy in type 1 diabetes mellitus show involvement of kallikrein-kinin system
Source: BMC Nephrol. 2017 Mar 30;18:112. doi: 10.1186/s12882-017-0519-4 (PMC5372325; doi:10.1186/s12882-017-0519-4)
Supplement: Supplementary file 2 — Variable importance in projection (VIP) scores for the 113 spots entering the PLS-DA model”. (DOCX 84 kb) [file 12882_2017_519_MOESM2_ESM.docx]

**
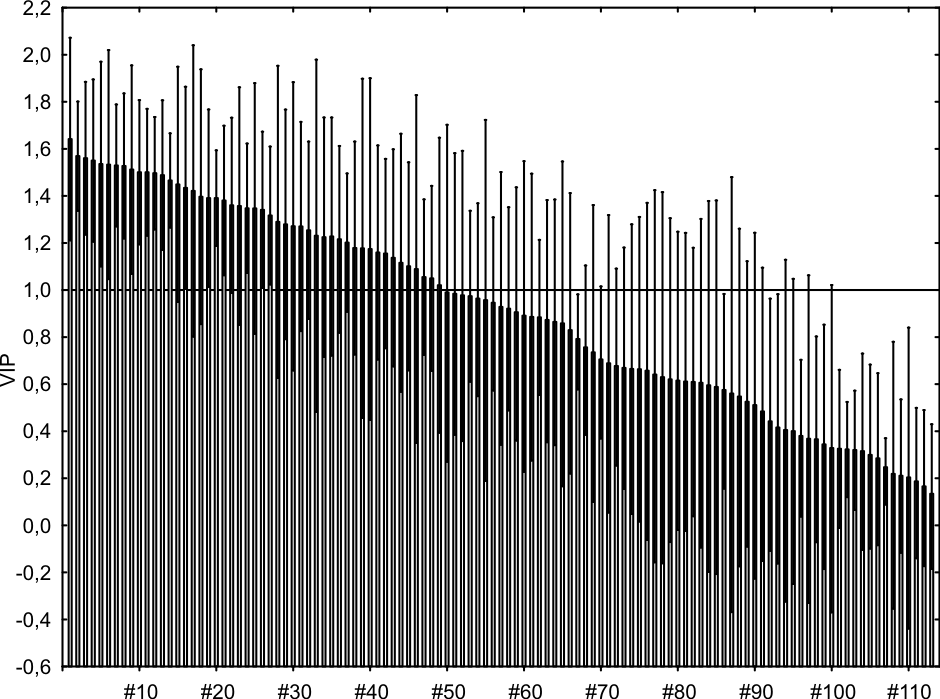
**

**Supplement 2 Variable importance in projection (VIP) scores for the 113 spots entering the PLS-DA model**

Variables (n = 49) reaching VIP score > 1.0 were selected for identification.
