# Supplementary material for: Early urinary biomarkers of diabetic nephropathy in type 1 diabetes mellitus show involvement of kallikrein-kinin system
Source: BMC Nephrol. 2017 Mar 30;18:112. doi: 10.1186/s12882-017-0519-4 (PMC5372325; doi:10.1186/s12882-017-0519-4)
Supplement: Supplementary file 3 — Combined MS and MS/MS sequence coverage of detected cleavage fragments”. (DOCX 22 kb) [file 12882_2017_519_MOESM3_ESM.docx]

1 MKLITILFLC SRLLLSLTQE SQSEEIDCND KDLFKAVDAA LK**KYNSQNQS**

51 **NNQFVLYR**IT EATK**TVGSDT FYSFK**YEIKE GDCPVQSGK**T WQDCEYKDAA**

101 KAATGECTAT VGKRSSTKFS VATQTCQITP AEGPVVTAQY DCLGCVHPIS

151 TQSPDLEPIL RHGIQYFNNN TQHSSLFMLN EVKRAQRQVV AGLNFRITYS

201 IVQTNCSK**EN FLFLTPDCKS LWNGDTGECT DNAYIDIQLR IASFSQNCDI**

251 **YPGKDFVQPP TKICVGCPRD IPTNSPELEE TLTHTITK**LN AENNATFYFK

301 IDNVKKARVQ VVAGK**KYFID FVARETTCSK ESNEELTESC ETKKLGQSLD**

351 **CNAEVYVVPW EKKIYPTVNC QPLGMISLMK RPPGFSPFR**S SRIGEIKEET

401 TVSPPHTSMA PAQDEERDSG KEQGHTRRHD WGHEKQRKHN LGHGHKHERD

451 QGHGHQRGHG LGHGHEQQHG LGHGHKFKLD DDLEHQGGHV LDHGHKHKHG

501 HGHGKHKNKG KKNGKHNGWK TEHLASSSED STTPSAQTQE KTEGPTPIPS

551 LAKPGVTVTF SDFQDSDLIA TMMPPISPAP IQSDDDWIPD IQIDPNGLSF

601 NPISDFPDTT SPKCPGRPWK SVSEINPTTQ MKESYYFDLT DGLS

**A**

1 MQTCPLAFPG HVSQALGTLL FLAASLSAQN EGWDSPICTE GVVSVSWGEN

51 TVMSCNISNA FSHVNIKLR**A HGQESAIFNE VAPGYFSR**DG WQLQVQGGVA

101 QLVIKGAR**DS HAGLYMWHLV GHQR**NNRQVT LEVSGAEPQS APDTGFWPVP

151 AVVTAVFILL VALVMFAWYR CRCSQQRREK KFFLLEPQMK VAALRAGAQQ

201 GLSRASAELW TPDSEPTPRP LALVFKPSPL GALELLSPQP LFPYAADP

**B**

1 MGWRAAGALL LALLLHGRLL AVTHGLRAYD GLSLPEDIET VTASQMRWTH

51 SYLSDDEDML ADSISGDDLG SGDLGSGDFQ MVYFRALVNF TRSIEYSPQL

101 EDAGSREFRE VSEAVVDTLE SEYLKIPGDQ VVSVVFIKEL DGWVFVELDV

151 GSEGNADGAQ IQEMLLRVIS SGSVASYVTS PQGFQFRRLG TVPQFPRACT

201 EAEFACHSYN ECVALEYRCD RRPDCRDMSD ELNCEEPVLG ISPTFSLLVE

251 TTSLPPRPET TIMRQPPVTH APQPLLPGSV RPLPCGPQEA ACRNGHCIPR

301 DYLCDGQEDC EDGSDELDCG PPPPCEPNEF PCGNGHCALK LWRCDGDFDC

351 EDRTDEANCP TKRPEEVCGP TQFRCVSTNM CIPASFHCDE ESDCPDRSDE

401 FGCMPPQVVT PPRESIQASR GQTVTFTCVA IGVPTPIINW RLNWGHIPSH

451 PRVTVTSEGG RGTLIIRDVK ESDQGAYTCE AMNARGMVFG IPDGVLELVP

501 QRGPCPDGHF YLEHSAACLP CFCFGITSVC QSTRRFRDQI RLRFDQPDDF

551 KGVNVTMPAQ PGTPPLSSTQ LQIDPSLHEF QLVDLSRRFL VHDSFWALPE

601 QFLGNKVDSY GGSLRYNVRY ELARGMLEPV QRPDVVLMGA GYRLLSRGHT

651 PTQPGALNQR QVQFSEEHWV HESGRPVQRA ELLQVLQSLE AVLIQTVYNT

701 KMASVGLSDI AMDTTVTHAT SHGRAHSVEE CRCPIGYSGL SCESCDAHFT

751 RVPGGPYLGT CSGCNCNGHA SSCDPVYGHC LNCQHNTEGP QCNKCKAGFF

801 GDAMKATATS CRPCPCPYID ASRRFSDTCF LDTDGQATCD ACAPGYTGRR

851 CESCAPGYEG NPIQPGGKCR PVNQEIVRCD ERGSMGTSGE ACRCKNNVVG

901 RLCNECADGS FHLSTRNPDG CLKCFCMGVS RHCTSSSWSR AQLHGASEEP

951 GHFSLTNAAS THTTNEGIFS PTPGELGFSS FHRLLSGPYF WSLPSRFLGD

1001 KVTSYGGELR FTVTQRSQPG STPLHGQPLV VLQGNNIILE HHVAQEPSPG

1051 QPSTFIVPFR EQAWQRPDGQ PATREHLLMA LAGIDTLLIR ASYAQQPAES

1101 RVSGISMDVA VPEETGQDPA LEVEQCSCPP GYRGPSCQDC DTGYTRTPSG

1151 LYLGTCERCS CHGHSEACEP ETGACQGCQH HTEGPRCEQC QPGYYGDAQR

1201 GTPQDCQLCP CYGDPAAGQA AHTCFLDTDG HPTCDACSPG HSGRHCERCA

1251 PGYYGNPSQG QPCQRDSQVP GPIGCNCDPQ GSVSSQCDAA GQCQCKAQVE

1301 GLTCSHCRPH HFHLSASNPD GCLPCFCMGI TQQCASSAYT RHLISTHFAP

1351 GDFQGFALVN PQRNSRLTGE FTVEPVPEGA QLSFGNFAQL GHESFYWQLP

1401 ETYQGDKVAA YGGKLRYTLS YTAGPQGSPL SDPDVQITGN NIMLVASQPA

1451 LQGPERRSYE IMFREEFWRR PDGQPATREH LLMALADLDE LLIRATFSSV

1501 PLAASISAVS LEVAQPGPSN RPRALEVEEC RCPPGYIGLS CQDCAPGYTR

1551 TGSGLYLGHC ELCECNGHSD LCHPETGACS QCQHNAAGEF CELCAPGYYG

1601 DATAGTPEDC QPCACPLTNP ENMFSRTCES LGAGGYRCTA CEPGYTGQYC

1651 EQCGPGYVGN PSVQGGQCLP ETNQAPLVVE VHPARSIVPQ GGSHSLRCQV

1701 SGSPPHYFYW SREDGRPVPS GTQQRHQGSE LHFPSVQPSD AGVYICTCRN

1751 LHQSNTSRAE LLVTEAPSKP ITVTVEEQRS QSVRPGADVT FICTAKSKSP

1801 AYTLVWTRLH NGKLPTRAMD FNGILTIRNV QLSDAGTYVC TGSNMFAMDQ

1851 GTATLHVQAS GTLSAPVVSI HPPQLTVQPG QLAEFRCSAT GSPTPTLEWT

1901 GGPGGQLPAK AQIHGGILRL PAVEPTDQAQ YLCRAHSSAG QQVARAVLHV

1951 HGGGGPRVQV SPERTQVHAG RTVRLYCRAA GVPSATITWR KEGGSLPPQA

2001 RSERTDIATL LIPAITTADA GFYLCVATSP AGTAQARIQV VVLSASDASP

2051 PPVKIESSSP SVTEGQTLDL NCVVAGSAHA QVTWYRRGGS LPPHTQVHGS

2101 RLRLPQVSPA DSGEYVCRVE NGSGPKEASI TVSVLHGTHS GPSYTPVPGS

2151 TRPIRIEPSS SHVAEGQTLD LNCVVPGQAH AQVTWHKRGG SLPARHQTHG

2201 SLLRLHQVTP ADSGEYVCHV VGTSGPLEAS VLVTIEASVI PGPIPPVRIE

2251 SSSSTVAEGQ TLDLSCVVAG QAHAQVTWYK RGGSLPARHQ VRGSRLYIFQ

2301 ASPADAGQYV CRASNGMEAS ITVTVTGTQG ANLAYPAGST QPIRIEPSSS

2351 QVAEGQTLDL NCVVPGQSHA QVTWHKRGGS LPVRHQTHGS LLRLYQASPA

2401 DSGEYVCRVL GSSVPLEASV LVTIEPAGSV PALGVTPTVR IESSSSQVAE

2451 GQTLDLNCLV AGQAHAQVTW HKRGGSLPAR HQVHGSRLRL LQVTPADSGE

2501 YVCRVVGSSG TQEASVLVTI QQRLSGSHSQ GVAYPVRIES SSASLANGHT

2551 LDLNCLVASQ APHTITWYKR GGSLPSRHQI VGSRLRIPQV TPADSGEYVC

2601 HVSNGAGSRE TSLIVTIQGS GSSHVPSVSP PIRIESSSPT VVEGQTLDLN

2651 CVVARQPQAI ITWYKRGGSL PSRHQTHGSH LRLHQMSVAD SGEYVCRANN

2701 NIDALEASIV ISVSPSAGSP SAPGSSMPIR IESSSSHVAE GETLDLNCVV

2751 PGQAHAQVTW HKRGGSLPSH HQTRGSRLRL HHVSPADSGE YVCRVMGSSG

2801 PLEASVLVTI EASGSSAVHV PAPGGAPPIR IEPSSSRVAE GQTLDLKCVV

2851 PGQAHAQVTW HKRGGNLPAR HQVHGPLLRL NQVSPADSGE YSCQVTGSSG

2901 TLEASVLVTI EPSSPGPIPA PGLAQPIYIE ASSSHVTEGQ TLDLNCVVPG

2951 QAHAQVTWYK RGGSLPARHQ THGSQLRLHL VSPADSGEYV CRAASGPGPE

3001 QEASFTVTVP PSEGSSYRLR SPVISIDPPS STVQQGQDAS FKCLIHDGAA

3051 PISLEWKTRN QELEDNVHIS PNGSIITIVG TRPSNHGTYR CVASNAYGVA

3101 QSVVNLSVHG PPTVSVLPEG PVWVKVGKAV TLECVSAGEP RSSARWTRIS

3151 STPAKLEQRT YGLMDSHAVL QISSAKPSDA GTYVCLAQNA LGTAQKQVEV

3201 IVDTGAMAPG APQVQAEEAE LTVEAGHTAT LRCSATGSPA PTIHWSKLRS

3251 PLPWQHRLEG DTLIIPRVAQ QDSGQYICNA TSPAGHAEAT IILHVESPPY

3301 ATTVPEHASV QAGETVQLQC LAHGTPPLTF QWSRVGSSLP GRATARNELL

3351 HFERAAPEDS GRYRCRVTNK VGSAEAFAQL LVQGPPGSLP ATSIPAGSTP

3401 TVQVTPQLET KSIGASVEFH CAVPSDRGTQ LRWFKEGGQL PPGHSVQDGV

3451 LRIQNLDQSC QGTYICQAHG PWGKAQASAQ LVIQALPSVL INIRTSVQTV

3501 VVGHAVEFEC LALGDPKPQV TWSKVGGHLR PGIVQSGGVV RIAHVELADA

3551 GQYRCTATNA AGTTQSHVLL LVQALPQISM PQEVRVPAGS AAVFPCIASG

3601 YPTPDISWSK LDGSLPPDSR LENNMLMLPS VRPQDAGTYV CTATNRQGKV

3651 KAFAHLQVPE RVVPYFTQTP YSFLPLPTIK DAYRKFEIKI TFRPDSADGM

3701 LLYNGQKRVP GSPTNLANRQ PDFISFGLVG GRPEFRFDAG SGMATIRHPT

3751 PLALGHFHTV TLLRSLTQGS LIVGDLAPVN GTSQGKFQGL DLNEELYLGG

3801 YPDYGAIPKA GLSSGFIGCV RELRIQGEEI VFHDLNLTAH GISHCPTCRD

3851 RPCQNGGQCH DSESSSYVCV CPAGFTGSRC EHSQALHCHP EACGPDATCV

3901 NRPDGRGYTC RCHLGRSGLR CEEGVTVTTP SLSGAGSYLA LPALTNTHHE

3951 LRLDVEFKPL APDGVLLFSG GKSGPVEDFV SLAMVGGHLE FRYELGSGLA

4001 VLRSAEPLAL GRWHRVSAER LNKDGSLRVN GGRPVLRSSP GKSQGLNLHT

4051 LLYLGGVEPS VPLSPATNMS AHFRGCVGEV SVNGKRLDLT YSFLGSQGIG

4101 QCYDSSPCER QPCQHGATCM PAGEYEFQCL CRDGFKGDLC EHEENPCQLR

4151 EPCLHGGTCQ GTRCLCLPGF SGPRCQQGSG HGIAESDWHL EGSGGNDAPG

4201 QYGAYFHDDG FLAFPGHVFS R**SLPEVPETI ELEVR**TSTAS GLLLWQGVEV

4251 GEAGQGKDFI SLGLQDGHLV FR**YQLGSGEA RLVSEDPIND GEWHR**VTALR

4301 EGR**RGSIQVD GEELVSGR**SP GPNVAVNAK**G SVYIGGAPDV ATLTGGR**FSS

4351 GITGCVKNLV LHSARPGAPP PQPLDLQHRA QAGANTRPCP S

**C**

1 MKPPRPVRTC SKVLVLLSLL AIHQTTTAEK NGIDIYSLTV DSRVSSRFAH

51 TVVTSRVVNR ANTVQEATFQ MELPKKAFIT NFSMIIDGMT YPGIIKEKAE

101 AQAQYSAAVA KGKSAGLVKA TGRNMEQFQV SVSVAPNAKI TFELVYEELL

151 KRRLGVYELL LKVRPQQLVK HLQMDIHIFE PQGISFLETE STFMTNQLVD

201 ALTTWQNKTK AHIRFKPTLS QQQKSPEQQE TVLDGNLIIR YDVDRAISGG

251 SIQIENGYFV HYFAPEGLTT MPKNVVFVID KSGSMSGRKI QQTREALIKI

301 LDDLSPRDQF NLIVFSTEAT QWRPSLVPAS AENVNKARSF AAGIQALGGT

351 NINDAMLMAV QLLDSSNQEE RLPEGSVSLI ILLTDGDPTV GETNPRSIQN

401 NVREAVSGRY SLFCLGFGFD VSYAFLEKLA LDNGGLARRI HEDSDSALQL

451 QDFYQEVANP LLTAVTFEYP SNAVEEVTQN NFRLLFKGSE MVVAGKLQDR

501 GPDVLTATVS GKLPTQNITF QTESSVAEQE AEFQSPKYIF HNFMERLWAY

551 LTIQQLLEQT VSASDADQQA LRNQALNLSL AYSFVTPLTS MVVTKPDDQE

601 QSQVAEKPME GESRNRNVHS GSTFFKYYLQ GAKIPKPEAS FSPRRGWNRQ

651 AGAAGSRMNF RPGVLSSRQL GLPGPPDVPD HAAYHPFRRL AILPASAPPA

701 TSNPDPAVSR VMNMKIEETT MTTQTPAPIQ APSAILPLPG QSVERLCVDP

751 RHRQGPVNLL SDPEQGVEVT GQYEREKAGF SWIEVTFKNP LVWVHASPEH

801 VVVTRNRRSS AYKWK**ETLFS VMPGLK**MTMD KTGLLLLSDP DKVTIGLLFW

851 DGRGEGLRLL LRDTDRFSSH VGGTLGQFYQ EVLWGSPAAS DDGRR**TLRVQ**

901 **GNDHSATR**ER RLDYQEGPPG VEISCWSVEL

**D**

1 MILSLLFSLG GPLGWGLLGA WAQASSTSLS DLQSSRTPGV WKAEAEDTGK

51 DPVGRNWCPY PMSKLVTLLA LCKTEKFLIH SQQPCPQGAP DCQKVKVMYR

101 MAHKPVYQVK QKVLTSLAWR CCPGYTGPNC EHHDSMAIPE PADPGDSHQE

151 PQDGPVSFKP GHLAAVINEV EVQQEQQEHL LGDLQNDVHR VADSLPGLWK

201 ALPGNLTAAV MEANQTGHEF PDRSLEQVLL PHVDTFLQVH FSPIWRSFNQ

251 SLHSLTQAIR NLSLDVEANR QAISRVQDSA VARADFQELG AKFEAKVQEN

301 TQRVGQLRQD VEDRLHAQHF TLHRSISELQ ADVDTKLKRL HKAQEAPGTN

351 GSLVLATPGA GARPEPDSLQ ARLGQLQRNL SELHMTTARR EEELQYTLED

401 MRATLTRHVD EIKELYSESD ETFDQISKVE RQVEELQVNH TALRELRVIL

451 MEKSLIMEEN KEEVERQLLE LNLTLQHLQG GHADLIKYVK DCNCQKLYLD

501 LDVIREGQRD ATRALEETQV SLDERRQLDG SSLQALQNAV DAVSLAVDAH

551 KAEGERARAA TSRLRSQVQA LDDEVGALKA AAAEARHEVR QLHSAFAALL

601 EDALRHEAVL AALFGEEVLE EMSEQTPGPL PLSYEQIRVA LQDAASGLQE

651 QALGWDELAA RVTALEQASE PPRPAEHLEP SHDAGREEAA TTALAGLARE

701 LQSLSNDVKN VGRCCEAEAG AGAASLNASL HGLHNALFAT QRSLEQHQRL

751 FHSLFGNFQG LMEANVSLDL GKLQTMLSRK GKKQQKDLEA PRKRDKK**EAE**

801 **PLVDIR**VTGP VPGALGAALW EAGSPVAFYA SFSEGTAALQ TVKFNTTYIN

851 IGSSYFPEHG YFRAPERGVY LFAVSVEFGP GPGTGQLVFG GHHRTPVCTT

901 GQGSGSTATV FAMAELQKGE RVWFELTQGS ITKRSLSGTA FGGFLMFKT

**E**

**Supplement 3 Combined MS and MS/MS sequence coverage of detected cleavage fragments**

Sequences identified by MS are highlighted by underscore.

**A** Spot containing kininogen-1 extended over a considerable interval of pI and molecular weights suggesting presence of multiple forms or cleavage fragments. Material for MS analyses was excised from several parts of the spot’s area.

kininogen-1 heavy chain: [19 – 380](http://www.uniprot.org/blast/?about=P01042%5b19-380%5d)

bradykinin: [381 – 389](http://www.uniprot.org/blast/?about=P01042%5b381-389%5d)

kininogen light chain: [390 – 644](http://www.uniprot.org/blast/?about=P01042%5b390-644%5d)

**B** Secreted and transmembrane protein 1

extracellular domain: 29 – 145

transmembrane domain: 146 – 166

cytoplasmic domain: 167 - 248

**C** Basement membrane-specific heparan sulfate proteoglycan core protein

Endorepellin: 3687 – 4391

LG3 peptide: [4197 – 4391](http://www.uniprot.org/blast/?about=P98160%5b4197-4391%5d)

**D** Inter-alpha-trypsin inhibitor heavy chain H4

70 kDa inter-alpha-trypsin inhibitor heavy chain H4: [29 – 661](http://www.uniprot.org/blast/?about=Q14624%5b29-661%5d)

Potentially active peptide (bradykinin-like): [662 – 688](http://www.uniprot.org/blast/?about=Q14624%5b662-688%5d)

35 kDa inter-alpha-trypsin inhibitor heavy chain H4: [689 – 930](http://www.uniprot.org/blast/?about=Q14624%5b689-930%5d)

**E** multimerin 2

C1q domain: [821-949](http://web.expasy.org/cgi-bin/compute_pi/pi_tool1?Q9H8L6@821-949@average)
